# Functional Evaluation and Nephrotoxicity Assessment of Human Renal Proximal Tubule Cells on a Chip

**DOI:** 10.3390/bios12090718

**Published:** 2022-09-03

**Authors:** Bolin Jing, Lei Yan, Jiajia Li, Piaopiao Luo, Xiaoni Ai, Pengfei Tu

**Affiliations:** 1 Key Laboratory of Natural and Biomimetic Drugs, School of Pharmaceutical Sciences, Peking University, Beijing 100191, China; 2 R&D Department Beijing Daxiang Biotech, No. 80 Xingshikou Road, Beijing 100195, China

**Keywords:** human renal proximal tubule cells, integrated biomimetic array chip, transporter function, epithelial polarization, nephrotoxicity

## Abstract

An in vitro human renal proximal tubule model that represents the proper transporter expression and pronounced epithelial polarization is necessary for the accurate prediction of nephrotoxicity. Here, we constructed a high-throughput human renal proximal tubule model based on an integrated biomimetic array chip (iBAC). Primary human renal proximal tubule epithelial cells (hRPTECs) cultured on this microfluidic platform were able to form a tighter barrier, better transporter function and more sensitive nephrotoxicity prediction than those on the static Transwell. Compared with the human immortalized HK2 model, the hRPTECs model on the chip gained improved apical-basolateral polarization, barrier function and transporter expression. Polymyxin B could induce nephrotoxicity not only from the apical of the hRPTECs, but also from the basolateral side on the iBAC. However, other chemotherapeutic agents, such as doxorubicin and sunitinib, only induced nephrotoxicity from the apical surface of the hRPTECs on the iBAC. In summary, our renal proximal tubule model on the chip exhibits improved epithelial polarization and membrane transporter activity, and can be implemented as an effective nephrotoxicity-screening toolkit.

## 1. Introduction

Drug-induced kidney injury (DIKI), one of the common adverse drug effects, is frequently discovered during later phases of pharmaceutical development and in clinical practices [1]. It is a key contributing factor to acute renal failure (ARF), and is associated with approximately 19–25% of reported cases. Renal proximal tubule epithelial cell is the primary target of renal injury among the 20 different cell types in the human kidney, due to its specialized functions. Physiologically, proximal tubular epithelium expresses numerous solute transporters on its apical and basolateral membrane, and packs with abundant mitochondria to support these energy-intensive transporters [2]. This transportation system selectively reabsorbs crucial nutrients, ions and water from glomerulus ultrafiltrate, and actively excretes endogenous and exogenous wastes, toxins and drugs, from blood [3]. The frequent exposure to xenobiotics potentiates their intracellular accumulation in proximal tubule epithelial cells, which could disrupt the regular mitochondria functions or impair other crucial cellular processes and eventually cascade into acute necrosis of these tubular cells.

In the current drug research and development (R&D) pipeline, animal study remains the gold standard for preclinical nephrotoxicity evaluation by performing renal histopathology after drug administration [4]. However, it bears the drawback of inefficiency and high cost. Although animal models exhibit high specificity for nephrotoxicity in drug dosage or safety pharmacology investigations, low sensitivity is also present [5]. In addition, due to species differences, many lead compounds with better potential were rejected as a result of their nephrotoxicity in animals [6]. Therefore, the establishment of an in vitro model mimicking human renal physiology for nephrotoxicity evaluation can retain more promising lead compounds and reduce the failure rate of clinical trials [7].

Conventionally, in vitro studies are performed in petri dishes or Transwell inserts, under static conditions. However, renal proximal tubular epithelial cells (RPTECs) in vivo are subjected to persistent luminal fluid shear stress (FSS) and inverse osmotic gradient across the epithelium, which is absent from these culture platforms [8,9,10]. To recapitulate such microenvironment features, a novel technology named organ-on-a-chip (OOC) is introduced [11,12]. OOC is a specialized subtype of the microfluidic chip, which mimics functional units of human organs in vitro [13]. It supports the construction of a micro-physiological system that simulates the functional units of human organs in vitro [9,14]. Recent studies have shown that renal-proximal-tubule-on-a-chip (RPTOC), the OOC variant for renal proximal tubule, can achieve the characteristics of renal tissue and has great potential in renal disease modeling and drug-induced kidney injury (DIKI) assessment [15,16]. FSS was shown to play a critical role in improving the functionality and polarization of renal proximal tubule in vitro [17,18]. Vriend et al. demonstrated that FSS increased albumin uptake, P-gp efflux and cell elongation, however, this was not attributed to a mechanosensitive mechanism related to primary cilia in PTECs, but rather likely to microvilli present at the apical membrane [19]. Jang et al. discovered exposure of the epithelial monolayer to an apical fluid shear stress enhanced epithelial cell polarization and primary cilia formation compared to traditional Transwell culture systems [20]. Recent studies have demonstrated that the RPTOC that drives the fluid by a pump has significant advantages over the 2D static model, but the advantages of high throughput RPTOC that drives the fluid by gravity has still not been studied. The immortalized proximal tubule epithelial cell line of HK2 cells and primary proximal tubule epithelial cells are currently used for studies of renal functionality and nephrotoxicity [21,22]; however, the differences in the functionality and nephrotoxicity evaluation between the primary renal proximal tubular cells and the immortalized HK2 cells on the chip has never been systematically compared. Moreover, the apical-basolateral polarization of the renal proximal tubule epithelium is crucial for the proper spatial organization of membrane transporter and influence the accuracy of drug toxicity evaluation [2]; however, the apical- or basal-specific RPTE toxicity evaluation are still rarely reported.

In this work, we designed an integrated biomimetic array chip (iBAC) that can achieve fluid shear forces similar to those in vivo through the action of gravity with the help of a precision rocker. Cell viability, barrier function, transporter expression, and nephrotoxicity sensitivity of the hRPTECs cultured on the fluid iBAC and the static Transwell were compared. We also systematically compared the barrier function, polarization state, and membrane transporter function of primary human renal proximal tubular epithelial cells (hRPTECs) and human immortalized renal proximal tubular epithelial cells (HK2) on the iBAC. We further assessed the nephrotoxic effects of model drugs administrated from the apical and basal side of the model. We anticipate our model being a promising tool for nephrotoxic screening and study of toxicological mechanism.

## 2. Materials and Methods

### 2.1. The Design of the Integrated Biomimetic Array Chip

An integrated biomimetic array chip (iBAC) was used to establish the human renal proximal tubule model. The chip consisted of twenty-four functional units and each unit was composed of five layers. The 1st, 2nd and 4th layers of the chip were made of polystyrene, and prepared by machining. The 3rd layer was a polyethylene terephthalate porous membrane with a thickness of 12 μm and the 5th layer was a glass plate. The cross-section view of the device and thick dimensions are shown in Figure S1. All of the materials were assembled with double-sided adhesive. Double-sided adhesive tape made by acrylic medical grade adhesive was purchased from Shenzhen Motian Corp. (Shenzhen, China), and its thickness was 50 μm. We developed the chip and it is commercially available from Daxiang Biotech.

### 2.2. Cells Culture

The primary human renal proximal tubule epithelial cells(hRPTECs)from Lonza were cultured in a REGM culture medium (Lonza, Basel, Switzerland). The human kidney 2 cells (HK2) from ATCC were cultured in a DMEM medium (Life Technologies, Paisley, UK) containing 10% (*w*/*v*) of fetal bovine serum (Life Technologies, Paisley, UK). Penicillin (100 units/mL) and streptomycin (100 mg/mL) were added to all aforementioned media. All cells were cultured in a cell incubator with 5% CO_2_ at 37 °C.

### 2.3. Construction of Human Renal Proximal Tubules on the iBAC

After the microdevice was assembled, it was exposed to ultraviolet light for 30 min. The porous membrane was coated with collagen type I hydrogel at 37 °C for 2 h. The hRPTECs or HK2 cells (2 × 10^5^ cells/ cm^2^) were seeded on the lower microfluidic channel and incubated upside down at 37 °C for 2 h, allowing the seeded hRPTECs or HK2 cells to attach to the porous membrane surface. Then, 100 μL of the culture medium was added to each chamber and the device was placed on the iBAC Rocker (MR100110, Daxiang biotech, Beijing, China) for dynamic culture.

The fluid dynamics on the iBAC were simulated using COMSOL Multiphysics to verify and optimize the swing frequency and swing angle for desired shear stress. Finally, the relative stable fluid flow rate (180 μL/min) and shear force (0.22 dyne/cm^2^) could be achieved when the swing angle was 30 degrees and the swing frequency was 1 circle/min (Figure S2). The shear stress inside the channel was calculated by COMSOL Multiphysics simulation.

### 2.4. TEER Assay

Before the resistance measurement, the chip reservoir and channel were cleaned by PBS twice at room temperature. Subsequently, 100 μL PBS were added to the chip reservoir and probes of the resistance meter (MT100110, Daxiang biotech, Beijing, China) were equilibriated for 5 min. The measurement of the resistance value was performed by placing the probes (MT100111, Daxiang biotech, Beijing, China) vertically in the middle and right chambers of the chip. Each hole was measured three times and the average value was calculated.

### 2.5. Measurement of Paracellular Permeability

The barrier-forming capacity of the human renal proximal tubule was evaluated by measuring the apparent permeability (*P_app_*) of FITC-labeled dextrans (Merck, Darmstadt, Germany) with different molecular weights (4 kDa, 40 kDa) through the monolayer. The serum-free culture medium (100 μL) containing FITC-labeled dextrans (1 μM) was perfused through the microchannel of the bottom layer at 2 circles/min, and the blank serum-free culture medium (100 μL) was added to the intermediate reservoir. After two hours of dynamic absorption, the sample concentrations of FITC-dextrans were determined by quantifying the fluorescence levels (Ex = 495 nm, Em = 535 nm) using a microplate reader.

The efflux function of hRPTECs was evaluated by measuring the apparent permeability (*P_app_*) of Rhodamine 123 and DiOC2 (Merck, Darmstadt, Germany) with or without the corresponding inhibitor. The serum-free culture medium (100 μL) containing Rhodamine 123 (2 μM) or DiOC2 (10 μM) with or without inhibitors (Verapamil at 10 μM or Ko143 at 10 μM) was perfused through the microchannel of the bottom layer at 2 circles/min, and the blank serum-free culture medium (100 μL) was added to the intermediate reservoir. After two hours of dynamic absorption, the sample concentrations of Rhodamine 123 (Ex = 495 nm, Em = 535 nm) and DiOC2 (Ex = 470 nm, Em = 510 nm) were detected by using a microplate reader.

The active absorption function of hRPTECs was evaluated by measuring the apparent permeability (*P_app_*) of FITC-albumin (Merck, Darmstadt, Germany), 4-Di-1-ASP (ASP+, Thermo Fisher, Waltham, MA, USA), 5(6)-Carboxyfluorescein (5−CF, Thermo Fisher, Waltham, MA, USA). The serum-free culture medium (100 μL) containing FITC-albumin was perfused through the microchannel of the bottom layer at 2 circles/min, and the blank serum-free culture medium (100 μL) was added to the intermediate reservoir. The serum-free culture medium (100 μL) containing ASP+ or 5−CF was added to the intermediate reservoir, and the blank serum-free culture medium (100 μL) was perfused through the microchannel of the bottom layer at 2 circles/min. After two hours of dynamic absorption, the sample concentrations of FITC-albumin (Ex = 495 nm, Em = 535 nm), ASP+ (Ex = 474 nm, Em = 606 nm) and 5−CF (Ex = 490 nm, Em = 515 nm) were detected by using a microplate reader (BMG Labtech, Germany).

The *P_app_* was calculated using the equation below:

*P_app_* [cm/s] = (1/AC_0_)(dQ/dt)

where A = area of mass transfer, C_0_ = donor concentration of reagent in the upper medium, and dQ/dt = transmembrane transportation rate.

### 2.6. Morphological Studies

The morphological observation was done by following the standard protocol. After cultivation, hRPTECs or HK2 cells were fixed with 4% paraformaldehyde (Thermo Fisher, Waltham, MA, USA) for 15 min, and permeabilized with 0.1% Triton X-100 (Thermo Fisher, Waltham, MA, USA) in PBS for 10 min, followed by being blocked with 3% BSA in PBS for 30 min at room temperature. After that, cells were incubated with a ZO−1 monoclonal antibody (Thermo Fisher, Waltham, MA, USA), acetylated tubulin monoclonal antibody (Sigma, Darmstadt, Germany), villin monoclonal antibody (Thermo Fisher, Waltham, MA, USA) or Na/K ATPase monoclonal antibody (abcam, UK) at 10 mg/mL in a blocking buffer for 1 h at room temperature. After being washed with PBS, cells were incubated with a Goat anti-Rabbit IgG (H+L) Super clonal Secondary Antibody (Life Technologies, Paisley, UK) and Alexa Fluor^®^ 594 conjugate at a dilution of 1:1000 for 1 h at room temperature. All of the nuclei were stained with DAPI Invitrogen, Paisley, UK). All of the skeletal protein were stained with F-actin (Invitrogen, Paisley, UK). Images were taken under an inverted laser scanning confocal microscope.

### 2.7. Quantitative Real-Time PCR

RNA isolation was performed on the hRPTECs after seven days of culture on the iBAC using Tiangen’s RNEasy Mini kit (Tiangen, Beijing, China) and according to the manufacturer’s instructions. The total RNA from the kit was converted to cDNA using HiScript III RT SuperMix kit (Thermo Fisher, Waltham, MA, USA). RT-qPCR was performed using ChamQ Universal SYBR qPCR kit (Vazyme, Nanjing, China) for the different transporters with GAPDH as the housekeeping gene.

### 2.8. Analysis of Cell Viability on the iBAC

The Live/Dead kit (Thermo Fisher, Waltham, MA, USA), LDH kit (Life Technologies, Paisley, UK) and CellTiter-Glo (Promega, Madison, WI, USA) were used to evaluate cell viability on the iBAC. These assays were performed on test compound-treated monolayers following the manufacturer’s instructions. The assay protocols are described briefly here. In the live/dead cell assay, the cells were incubated with the live/dead cell imaging reagents for 15–30 min at 37 °C and observed under a fluorescent microscope. The viable cells were stained green, while the dead ones were stained red. In the CellTiter-Glo luminescent cell viability assay, the cells were incubated with the CellTiter-Glo reagents for 30 min at room temperature and detected with a microplate reader. For the LDH assay, 50 μL of medium from each well of the treatment plates was transferred into fresh 96-well plates and an equal volume of LDH reaction mixture was added. The samples were incubated at room temperature for 30 min before the reaction was stopped by adding 50 μL of stop solution. The absorbance was then taken at 490 nm and 680 nm using a microplate reader (BMG Labtech, Germany).

### 2.9. Data Analysis and Quantification

All data were analyzed by averaging the values of at least three microfluidic units, with each unit representing one independent experiment. All statistical analyses were performed using GraphPad Prism software. The statistical significance of the data was tested using the *t*-test for two data sets or ordinary one-way ANOVA and Dunnett’s multiple comparison test for more than two groups unless otherwise specified. All results and error bars in this article were represented as mean ± SD.

## 3. Results

### 3.1. Characteristics of Human Renal Proximal Tubule Model on the iBAC

**Figure 1:**
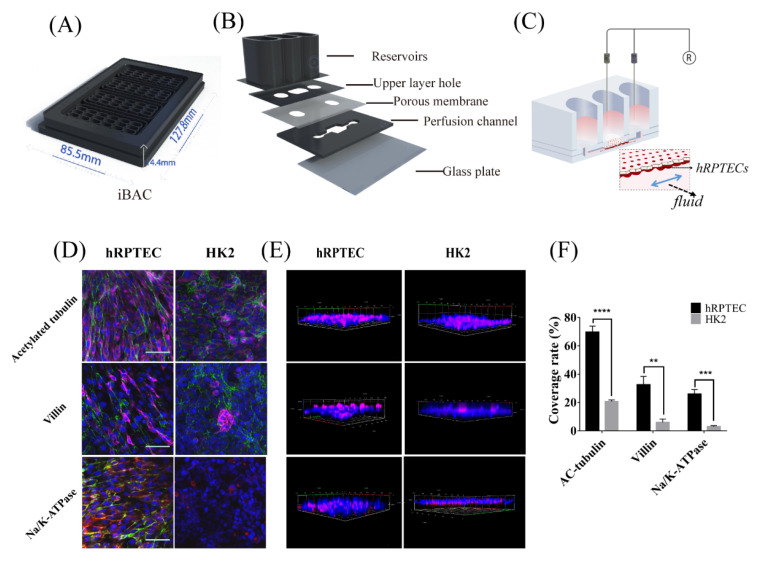
hRPTECs model on the integrated biomimetic array chip (iBAC). (**A**) Picture of an integrated biomimetic array chip containing 24 units. (**B**) Schematic exploded view of a unit on the iBAC. (**C**) Diagram showing the hRPTECs cultured on the iBAC. (**D**,**E**) Immunofluorescence micrographs of the hRPTECs and HK2 cells cultured on the iBAC for 4 days labeled with acetylated tubulin (apical), villin (apical) and Na/K-ATPase (basolateral), (bar, 100 µm). (**F**) The coverage rate of the acetylated tubulin, villin and Na/K-ATPase on the hRPTECs and HK2 cells cultured on the iBAC on day 4. ** *p* < 0.01, *** *p* < 0.001, **** *p* < 0.0001.

An integrated biomimetic array chip (iBAC) was designed to establish the proximal renal tubule model. The overall size of the iBAC was similar to a traditional 96-well plate with a length of 12.7 cm, a width of 8.5 cm and a height of 1.7 cm (Figure 1A). The iBAC consisted of 24 functional units. Each unit was composed of five layers, including the 1st layer with reservoirs, the 2nd layer with an upper layer hole, the 3rd permeability layer with a 0.4 µm PET porous membrane, the 4th layer with a perfusion channel to provide bionic fluid shear stress for proximal renal tubule cells, and the 5th layer with a glass plate at a thickness of 0.13 mm (Figure 1B). Human primary renal proximal tubule epithelial cells (hRPTECs) and immortalized HK2 cells were seeded onto the basal side of the porous membrane and exposed to the fluid (Figure 1C). The iBAC was set on a shaker to precisely control the shear stress and improve nutrition supply in the perfusion channel by optimizing the shaking angle and frequency (Figure S3).

The proximal renal tubule cells could be continuously cultured on the iBAC for more than one week. Herein, we compared the polarization status of the hRPTECs model with the HK2 model after one week of dynamic cultivation. The cilia and microvilli could sense shear forces and protect cells from damage. Immunofluorescence characterization of acetyl-α-tubulin and villin showed that the expression level of the cilia and microvilli on the apical side of the hRPTECs increased more than that on the HK2 cells (Figure 1D,E). Na/K-ATPase was expressed on the basal side of the proximal renal tubule, which was the main functional protein for Na^+^ reabsorption in the body. On the iBAC, the expression level of the Na/K-AtPase on the basal side of the hRPTECs was also higher than that on the HK2 cells (Figure 1D,E). Statistical analysis of the coverage rate of the acetylated tubulin, villin and Na/K-ATPase further showed the higher expressions of the proteins on the hRPTECs model in comparison to the HK2 model cultured on the iBAC (Figure 1F).

### 3.2. The Barrier Function of Human Renal Proximal Tubule Model on the iBAC

**Figure 2:**
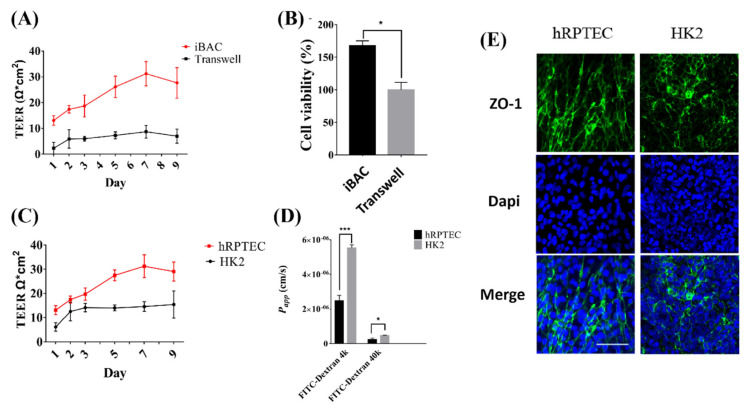
The barrier function of hRPTECs model on the iBAC. (**A**) TEER values of the hRPTECs cultured on the iBAC and Transwell during 9 days culture. (**B**) Cells viability of the hRPTECs cultured on the iBAC and Transwell on day 4. (**C**) TEER values of the hRPTECs and HK2 models cultured on the iBAC for 9 days. (**D**) The *P_app_* values of FITC-labeled dextran (4 kDa, 40 kDa) through the hRPTECs and HK2 models cultured on the iBAC for 4 days. (**E**) Immunofluorescence micrographs of hRPTECs and HK2 cells cultured on the iBAC for 4 days labeled with ZO−1 (Green) and nuclear labeled with Dapi (Blue), (bar, 100 µm). * *p* < 0.05, *** *p* < 0.001.

The barrier function of the hRPTECs prevented the diffusion of most compounds into the blood. Firstly, the barrier function of the hRPTECs model cultured on the iBAC and the static Transwell was compared. The hRPTECs model cultured on the iBAC had a denser morphology and higher cell viability than that on the Transwell (Figure 2A,B). By monitoring the TEER values for 9 days, the maximum TEER value of the hRPTECs model cultured on the iBAC could reach 30 Ω × cm^2^, while that on the static Transwell was only 8 Ω × cm^2^. Moreover, the maximum TEER value of the HK2 model cultured on the iBAC could reach 12 Ω × cm^2^, which was lower than the hRPTECs mode on the iBAC (Figure 2C). The *P_app_* values of FITC-labeled dextrans (4 kDa and 40 kDa) through the hRPTECs model on the iBACwere also lower than that of the HK2 model on the iBAC (Figure 2D). The intercellular junction proteins ZO−1 was an important markers of barrier compactness. Compared with the HK2 model, the expression of intercellular junction proteins was significantly higher on the hRPTECs model that were dynamically cultured on the iBAC (Figure 2E). All these data indicated that the hRPTECs model had stronger barrier functions under the dynamic culture conditions on the iBAC.

### 3.3. The Active Absorption Function of Human Renal Proximal Tubule Model on the iBAC

**Figure 3:**
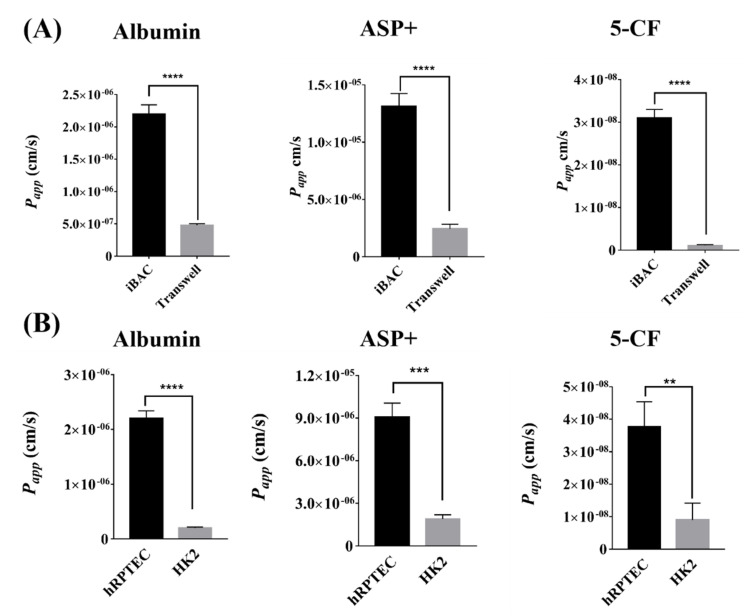
Active absorption function of hRPTECs model on the iBAC. (**A**) The *P_app_* value of FITC-labeled albumin, ASP+, or 5−CF through hRPTECs cultured on the iBAC and Transwell on day 4. (**B**) The *P_app_* value of FITC-labeled albumin, ASP+, or 5−CF through the hRPTECs and HK2 cells cultured on the iBAC on day 4. The data were obtained from two independent experiments with three replicates. ** *p* < 0.01, *** *p* < 0.001, **** *p* < 0.0001.

Membrane transporters in renal proximal tubules cells play an important role in drug absorption, accumulation and toxicity. The transport of albumin in renal proximal tubules is mainly mediated by Megalin/Cubilin. Here, we compared the absorption capacity of FITC-labeled albumin through the hRPTECs model cultured on the iBAC and the static Transwell. The *P_app_* value of the FITC-albumin through the hRPTECs on the iBAC reached 2 × 10^−6^ cm/s, whereas that on the Transwell was only 5 × 10^−7^ cm/s (Figure 3A). Moreover, the *P_app_* value of the FITC-albumin through the HK2 model on the iBAC was only 0.2 × 10^−6^ cm/s (Figure 3B). OCT2 is mainly expressed on the basal side of the proximal renal tubules that mediates organic cation transport, and ASP+ is one of the commonly used substrates of the OCT2 transporter. The *P_app_* value of the ASP+ through the hRPTECs on the iBAC was significantly higher than that on the Transwell (Figure 3A). Again, the *P_app_* value of the ASP+ through the hRPTECs model on the iBACwas also higher than that through the HK2 model on the iBAC, which were 9.2 × 10^−6^ cm/s and 2.3 × 10^−6^ cm/s, respectively (Figure 3B). OAT1 is also mainly expressed on the basal side of the proximal renal tubules that mediates organic anion transport. Herein, 5−CF was selected as the substrate to compare the OAT1 absorption ability through the hRPTECs cultured on the iBAC and the static Transwell. The results showed that the absorption efficiency of the OAT1 on the hRPTECs model cultured on the iBAC was higher than that on the Transwell, reaching 3.9 × 10^−8^ cm/s and 0.2 × 10^−8^ cm/s, respectively (Figure 3A). Moreover, the *P_app_* value of the 5−CF through the HK2 model on the iBAC was only 0.9 × 10^−8^ cm/s (Figure 3B). The relative mRNA expression of the Megalin, OCT2 and OAT1 was also compared between the hRPTECs model and the HK2 model cultured on the iBAC. The result showed that the mRNA expression of the Megalin, OCT2 and OAT1 on the hRPTECs were significantly higher than that on HK2 cells (Figure S4A). Therefore, the hRPTECs model showed improved active absorption function under the dynamic culture conditions on the iBAC.

### 3.4. The Efflux Transporter Function of Human Renal Proximal Tubule Model on the iBAC

**Figure 4:**
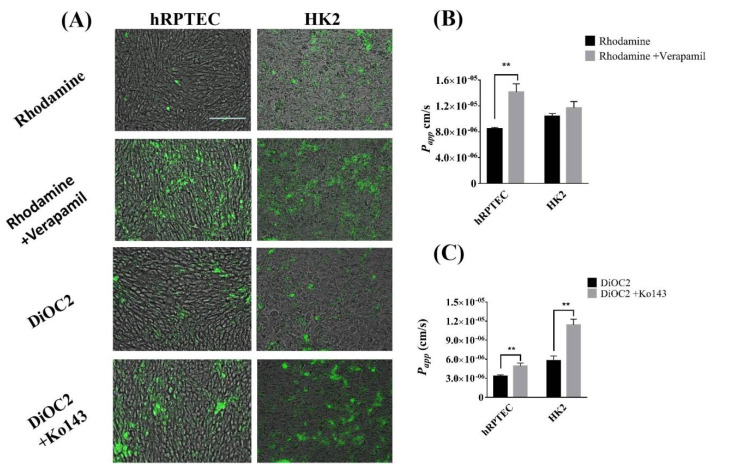
Efflux protein function of hRPTECs model on the iBAC. (**A**) Photographs of P−gp substrate of rhodamine 123 and BCRP substrate of DiOC2 accumulated in the hRPTECs and HK2 cells cultured on the iBAC for 4 days (bar, 200 µm). (**B**) The *P_app_* values of the rhodamine 123 through the hRPTECs and HK2 cells cultured on the iBAC for 4 days. (**C**) The *P_app_* values of the DiOC2 through the hRPTECs and HK2 cells cultured on the iBAC for 4 days. ** *p* < 0.01.

The ABC transporters of the renal proximal tubule cells include P-gp, BCRP and MRP1, 2 and 4, which can excrete most of the compounds back to the tubule and out of the body to avoid the nephrotoxicity caused by drug accumulation. To explore transporter-mediated efflux function between the hRPTECs model and the HK2 model on the iBAC, the cumulative effect of rhodamine 123 and DiOC2, known as substrates of the P-gp and BCRP, was investigated. We found that the cumulative amount of the rhodamine 123 and DiOC2 on the hRPTECs model and HK2 model was very low. Under the treatment of the P-gp protein inhibitor of verapamil and BCRP protein inhibitor of Ko143, the cumulative amounts of the two substrates on the two models were markedly boosted (Figure 4A). This indicated that the expression of the efflux proteins on the models. Next, the *P_app_* values of the rhodamine 123 and DiOC2 through the hRPTECs and HK2 models on the iBAC were quantified. The *P_app_* values of the rhodamine 123 through the hRPTECs model on the iBAC in the presence and absence of the verapamil were 5.6 × 10^−6^ cm/s and 1.3 × 10^−5^ cm/s, respectively. The inhibitor increased the permeability by 2.3 times (Figure 4B). Under the same conditions, the increased permeability on the HK2 model was only 1.2 times. Furthermore, the *P_app_* values of the DiOC2 through the hRPTECs model on the iBAC with or without the Ko143 were 4.1 × 10^−6^ cm/s and 4.6 × 10^−6^ cm/s, respectively. The inhibitor increased the permeability by 1.1 times (Figure 4C). Under the same conditions, the increased permeability on the HK2 model was reached 1.9 times. This indicated that the efflux function of the P-gp protein on the hRPTECs model was stronger than that on the HK2 model on the iBAC, whereas the efflux function of the BCRP on the hRPTECs model was similar to the HK2 model on the iBAC. Furthermore, the relative mRNA expression of the P-gp and MRP2 on the hRPTECs model was also higher than that on the HK2 model on the iBAC, whereas the expression of the BCRP on the hRPTECs model was similar to the HK2 model on the iBAC (Figure S4). In summary, the hRPTECs model on the iBAC showed superior to the HK2 model for kidney-related drug efflux and inhibitor research.

### 3.5. Nephrotoxicity of Cisplatin Mediated by OCT2 on the iBAC

**Figure 5:**
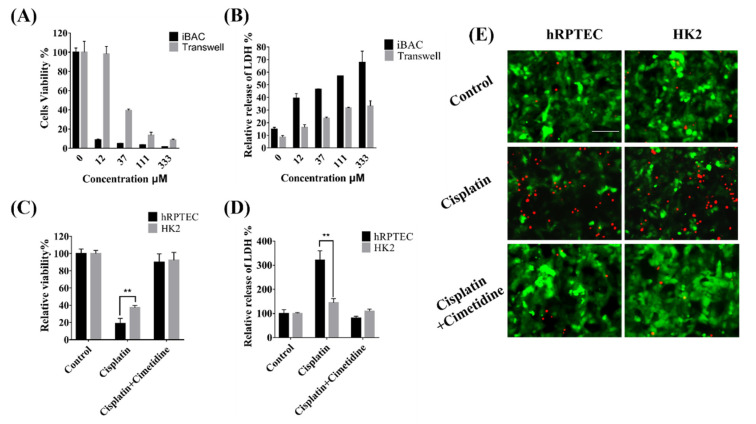
Evaluation of cisplatin nephrotoxicity on the iBAC. (**A**) Viability of the hRPTECs treated with cisplatin for 48 h. (**B**) Relative release of LDH from the hRPTECs treated with cisplatin for 48 h. (**C**) Relative viability of the hRPTECs and HK2 cells treated with the cisplatin in the presence and absence of the cimetidine on the iBAC for 24 h. (**D**) Relative release of LDH from the hRPTECs and HK2 cells treated with cisplatin in the presence and absence of the cimetidine on the iBAC for 24 h. (**E**) Photographs of the hRPTECs and HK2 cells treated with cisplatin in the presence and absence of the cimetidine on the iBAC, green fluorescence represents living cells and red fluorescence represents dead cells (bar, 100 µm). ** *p* < 0.01.

Cisplatin is the first metal complex with anticancer activity, and is a broad-spectrum anticancer drug in the clinic. Cisplatin can be absorbed by OCT2 and MATE1 in the proximal renal tubules, which result in cumulative nephrotoxicity. Firstly, the cisplatin was added onto the basal side of the hRPTECs on the iBAC and the static Transwell. Compared to that on the Transwell, the cisplatin on the iBAC induced lower cell viability and increased LDH release (Figure 5A,B). This result indicated that the dynamic culture conditions on the iBAC increased the sensitivity of the nephrotoxicity predictivity. Cimetidine is one of the OCT2 inhibitors. Furthermore, the cisplatin was added onto the basal side of the hRPTECs and HK2 cells on the iBAC. The number of dead cells increased significantly with the cisplatin alone, whereas the number of the dead cells decreased in the presence of the cimetidine (Figure 5E). By detection of the cell viability and LDH release, the viability of the hRPTECs and HK2 cells cultured on the iBAC in the cisplatin treatment group decreased to 19% and 38%, whereas the LDH release increased to 317% and 145%, respectively (Figure 5C,D). In the presence of the cimetidine, the ATP activity of the hRPTECs and HK2 cells maintained at 92% and 93%, whereas the LDH release maintained at 93% and 109%, respectively (Figure 5C,D). Compared to the HK2 model on the iBAC, the hRPTECs model on the iBAC also showed more sensitivity to the cisplatin exposure regarding significant differences in cell viability and LDH release. In conclusion, the cisplatin induced more severe proximal renal tubule toxicity on the dynamic culture of the hRPTECs on the iBAC, and the cimetidine significantly reversed the toxicity.

### 3.6. Toxicity Evaluation of Model Drugs on the Apical and Basal Side of hRPTECs on the iBAC

**Figure 6:**
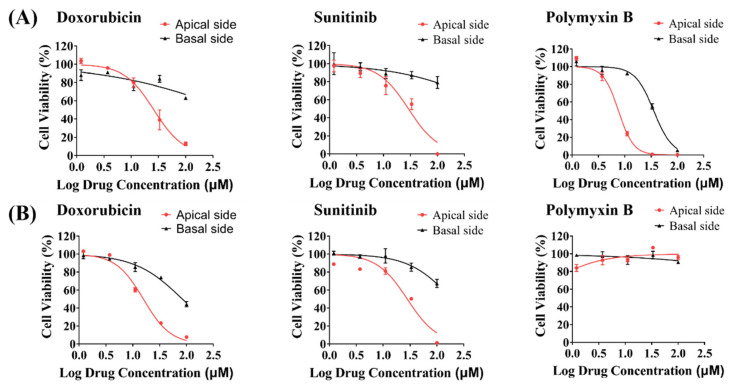
Nephrotoxicity evaluation of model drugs on the iBAC. Cell viability of the hRPTECs (**A**) and the HK2 cells (**B**) on the iBAC adminstrated with doxorubicin, sunitinib and polymyxin B for 48 h.

Chemotherapy drugs and antibiotics are commonly used clinical drugs that can cause nephrotoxicity. Herein, we evaluated the toxicity of doxorubicin, sunitinib and polymyxin B administrated from the apical and basal sides of the hRPTECs and HK2 cells. By detection of the cell viability after 48 h of drug treatment on the hRPTECs cultured on the iBAC, we found that all three drugs induced toxicity on the apical side of the hRPTECs, whereas only polymyxin B induced toxicity on the basal side of the hRPTECs (Figure 6A). We next detected the compound toxicity on the HK2 model (Figure 6B). Doxorubicin and sunitinib caused toxicity on the apical side of the HK2 cells, and the IC50 values were 14.16 μM, and 29 μM, respectively. Particularly, polymyxin B was not toxic on both sides of the HK2 cells on the iBAC. The apical- or basal-specific toxicity evaluation, as well as the mechanism of drug absorption and excretion, can be investigated by administration from the vascular cavity and urine cavity. In conclusion, the hRPTECs model showed higher sensitivity for nephrotoxicity prediction, and drug administration from the apical or basal side could be achieved on the iBAC.

## 4. Discussion

Worldwide, researchers are pushing the boundaries of nephrotoxicity prediction forward by improving the screening methodology, particularly with in vitro cell models, to minimize unnecessary risks in clinical settings and reduce the costs of pharmaceutical R&D [13,23]. The commonly used renal cell-based models are HK2 or MDCK cells cultured in multi-well plates, which cannot recreate the crucial physiological functions of the renal proximal tubule, and only allow restricted access to its basolateral surface for proper toxicity evaluation [24,25]. Compared with the static culture, the fluid shear stress on the microfluidic chip can significantly improve the morphology and functionality of the renal epithelial cells by promoting their apical-basolateral polarization and by enhancing the membrane transporter function [16,26]. In this study, we constructed a renal proximal tubule model on an integrated biomimetic array chip (iBAC) driven by bidirectional shaking, and systematically compared the cell growth, barrier function, transporter expression and drug toxicity of the hRPTECs on the iBAC and the static Transwell. To our knowledge, a systematical comparison of the functionality and nephrotoxicity evaluation between the primary cells (hRPTECs) and the immortalized cells (HK2 cells), as well as an investigation of the apical- or basal-specific nephrotoxicity, have never been performed. For the first time, the polarization status, barrier integrity and membrane transporter function of the hRPTECs model was systematically compared with the HK2 model under the dynamic culture on the iBAC. Finally, we achieved toxicity screening of drugs through apical or basal administration routines.

The in vitro PTOC is a promising platform for toxicology study, since it overcomes certain limitations of the animal model, such as high cost, low throughput and inconsistent prediction of human outcomes [27,28]. Most published works on the PTOC frequently implement cell lines including human HK2 cells and porcine LLC-PK cells [29]. However, the deficient expression of cell–cell junctional proteins and essential transporters impeded these cells from generating tight barriers with strong transport functions [10]. To overcome these limitations, our PTOC model was established using hRPTECs, and achieved considerable improvement in cellular polarization, barrier integrity and active transporter expression against the HK2 model.

Renal proximal tubule epithelium expressed transporters on both apical and basolateral membranes, which collectively mediate the elimination of drugs [30]. Meanwhile, endocytosis can occur at the basolateral and apical membrane of RPTECs in a receptor-mediated manner. For instance, albumin and other low molecular weight proteins from the glomerulus ultrafiltrate are re-uptaken via the apical membrane receptors of megalin and cubilin. The hRPTECs model on the iBAC improved albumin reabsorption compared to the HK2 model. Organic anion transporters (e.g., OAT1, OAT3, OATP4C1) and organic cation transporters (e.g., OCT2) located on the basolateral membrane, in conjunction with apically-expressed ABC efflux transporters (e.g., P-gp, BCRP, MRP2, MRP4), engage in the transcellular excretion of anionic and cationic drugs from systemic circulation. On the iBAC platform, the uptake efficiency of the OCT2 and OAT1 in the hRPTECs model was notably higher than that in the HK2 model. This indicated that the expression of the SLC22 transporter family on the immortalized proximal tubular cell line was insufficient [31]. Moreover, previous studies showed the supportive role of mechanical sweeping of microvilli on the function of transporters. Stimulated by the shear stress on the iBAC, the hRPTECs showed elevated density and height of microvilli, as well as higher microvilli-beating efficiency. The fluid flow on the iBAC also affected cellular structure modification and the functionality of apical efflux transporters. Consistent with our results, bidirectional fluidic culture condition could also boost the efflux functionality of the transporter within the HK2 cells [32].

Cisplatin, a classic nephrotoxic chemotherapeutic agent, is absorbed into RPTEC via the basolateral OCT2, copper transporter 1 (CTR1) and the less explored volume-regulated anion channel (VRAC) transporters [33]. The OCT2 transporter is expressed on the basolateral membrane of the PTEC and plays a key role in cisplatin uptake into the tubular cells [34]. The accumulation of the cisplatin in the hRPTECs and HK2 cells could trigger oxidative stress, cell apoptosis, necrosis, inflammation, vascular injury and endoplasmic reticulum stress. Compared with the HK2 model, the hRPTECs model on the iBAC showed decreased cell viability and increased LDH release after the cisplatin treatment. Cimetidine, as an OCT2 inhibitor, has been shown in previous studies to protect the RPTECs from cisplatin-induced cell injury in canine kidney cells and human embryonic kidney cells [18,20]. Our results show that cimetidine significantly inhibited the renal toxicity and LDH release on the hRPTECs model on the iBAC, however, there was no obvious inhibition of the LDH release on the HK2 model on the iBAC. More importantly, the hPRPTECs model on the iBAC were more sensitive to cisplatin exposure than that on the static Transwell, which was consistent with higher expression of the OCT2 transporter with the fluid stimulation.

Renal proximal tubule epithelium is a common site of drug-induced injury for its exposure to various intrinsic and xenobiotic chemicals from systemic circulation and its role in transporter-mediated drug clearance [35,36]. Doxorubicin, sunitinib and polymyxin B can induce clinical nephrotoxicity [6,37,38,39]. We evaluated the dose-response toxicity of these compounds using the hRPTECs and HK2 models on the iBAC. Drugs administrated from the apical or basal side could be achieved on the iBAC. For the first time, we discovered that the toxic effect of the polymyxin B can only be induced on the hRPTECs model, but not on the HK2 model. Previous studies showed that polymycin B triggered the release of kidney injury molecule 1 (KIM-1) and induced nephrotoxicity [21]. More importantly, the doxorubicin and sunitinib only induced nephrotoxicity on the apical side dosing. Therefore, the apical- or basal-specific toxicity evaluation, as well as the mechanism of drug absorption and excretion, can be investigated by administration from the vascular cavity and urine cavity on the iBAC.

## 5. Conclusions

In this study, we constructed a human renal proximal tubule model on an integrated biomimetic array chip (iBAC). The model on the chip offers some clear advantages over previously reported models. First, the iBAC was set on a shaker to precisely control the shear stress on the apical side of the hRPTECs. Commonly used microfluidic devices require external pumps and tubing to drive the unidirectional fluid, however, the iBAC achieved high throughput and an operationally simple methodology to drive the bidirectional flow by gravity. Second, compared to the static Transwell, the hRPTECs model on the iBAC with the bidirectional fluid exhibited a tighter barrier, improved transporter function and more sensitivity for predicting nephrotoxicity. Third, the differences in the functionality and nephrotoxicity evaluation between the primary cells (hRPTECs) and the immortalized cells (HK2 cells) on the chip were systematically compared. The performance of the hRPTECs model exhibited superior performance compared to the HK2 model in apical-basolateral polarization, barrier function, transporter expression and nephrotoxicity testing. Particularly, polymyxin B induced nephrotoxicity from both sides of the hRPTECs model on the iBAC, but not on the HK2 model. Finally, the iBAC offered two drug delivery methods for study of the apical- or basal-specific toxicity. The mechanism of drug absorption and excretion can be further investigated by drug administration from the vascular cavity and urine cavity. We anticipate that the established hRPTECs model on the iBAC will be a promising tool for nephrotoxic screening and the study of toxicological mechanisms.

## Data Availability

Not applicable.

## Supplementary Materials

The following supporting information can be downloaded at: https://www.mdpi.com/article/10.3390/bios12090718/s1, Figure S1: The cross-section view and thick dimensions of the device; Figure S2: Characterization of fluid flow rate and shear stress on the iBAC; Figure S3: Fluid flow on the iBAC drived by a rocker; Figure S4: The transporter expression of hRPTECs model on the iBAC.
