# Supplementary material for: Functional Evaluation and Nephrotoxicity Assessment of Human Renal Proximal Tubule Cells on a Chip
Source: Biosensors (Basel). 2022 Sep 3;12(9):718. doi: 10.3390/bios12090718 (PMC9496563; doi:10.3390/bios12090718)
Supplement: Supplementary file 1 [file biosensors-12-00718-s001.zip › biosensors-1865868-supplementary.pdf]

Supplementary Material

Functional Evaluation and Nephrotoxicity Assessment of Human Renal Proximal Tubule Cells on a Chip

Bolin Jing [1], Lei Yan [2], Jiajia Li [2], Piaopiao Luo [2], Xiaoni Ai [1,*] and Pengfei Tu [1,*]

[1] Key Laboratory of Natural and Biomimetic Drugs, School of Pharmaceutical Sciences, Peking University, Beijing, China[2]Beijing Daxiang Biotech, Beijing, China

* Correspondence: aixn@bjmu.edu.cn (X.A.); pengfeitu@bjmu.edu.cn (P.T.)

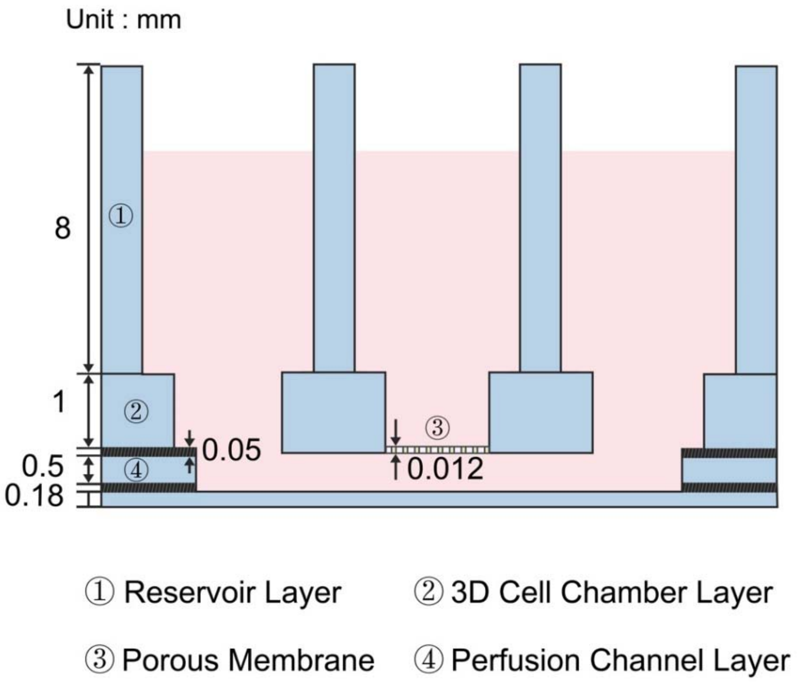

Supplementary Figure S1. The cross-section view and thick dimensions of the device.

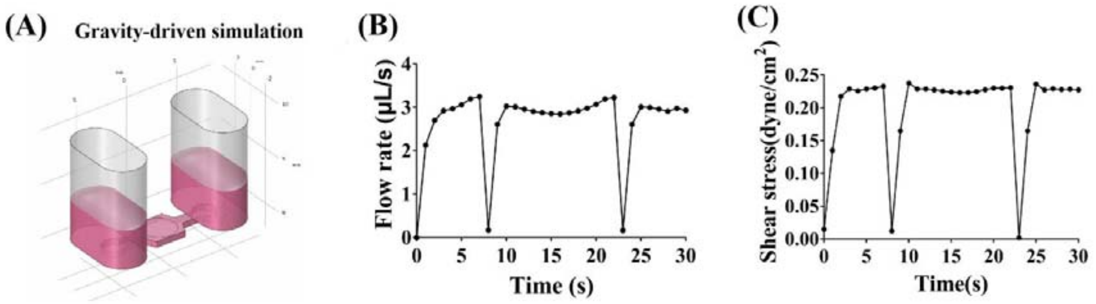

Supplementary Figure S2. Characterization of fluid flow rate and shear stress on the iBAC. (A) 3D view of the fluid distribution in the perfusion channel. Simulation of the fluid flow rate (B) and the shear stress (C) with the tilting time on the central area of the hRPTEC surface.

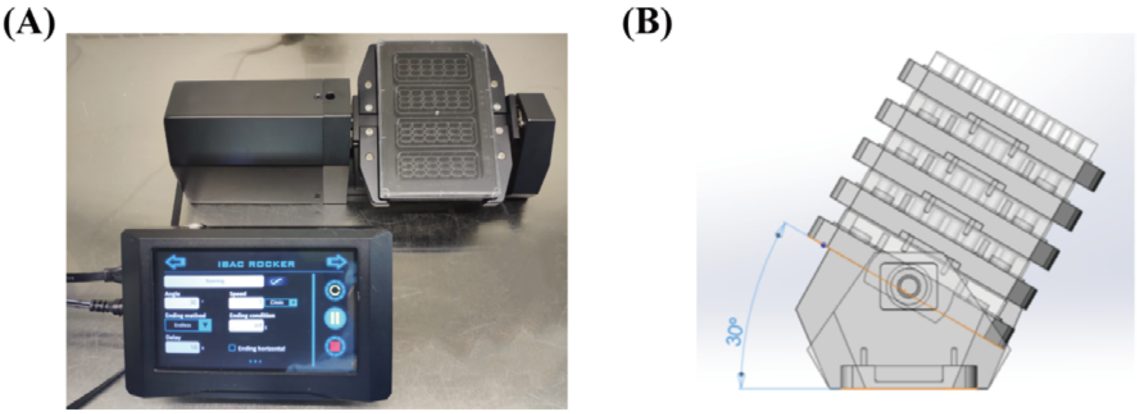

Supplementary Figure S3. Fluid flow on the iBAC drived by a rocker. The photo (A) and schematic diagram (B) of the shaker to drive the fluid flow on the iBAC.

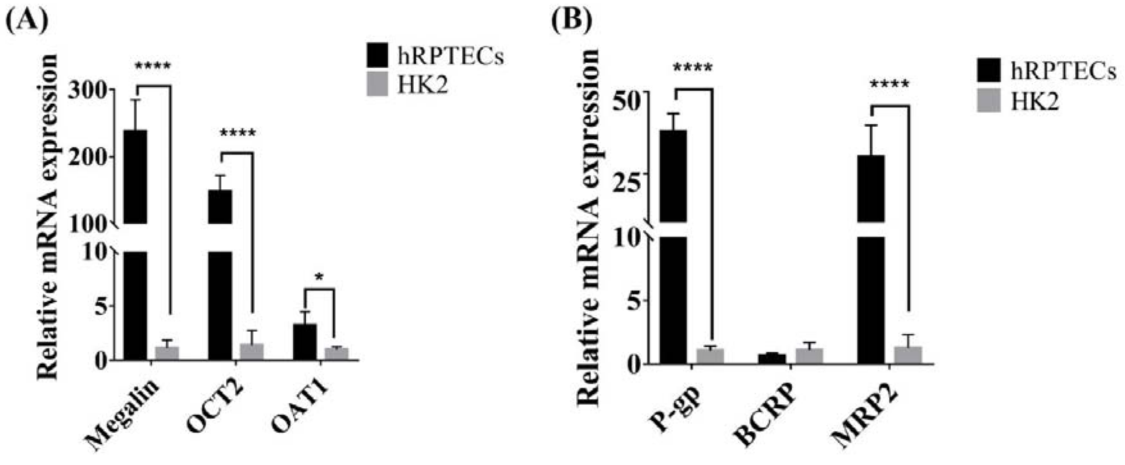

Supplementary Figure S4. The transporter expression of hRPTECs model on the iBAC. Comparison of the transporter function of the hRPTECs model cultured on the iBAC and Transwell on day 4. (A) mRNA levels of Megalin, OCT2 and OAT1 in the hRPTECs relative to the HK2 cells cultured on the iBAC on day 4. (B) mRNA levels of P-gp, BCRP and MRP2 on the hRPTECs model relative to the HK2 model cultured on the iBAC on day 4.
